# Occurrence of *MDR1* 1-delta mutation in herding dog breeds in Portugal

**DOI:** 10.3389/fvets.2022.990884

**Published:** 2022-10-03

**Authors:** Maria Cristina Barroso, Andreia Grilo, Sandra Aguiar, Frederico Aires da Silva, Berta São Braz

**Affiliations:** ^1^CIISA – Centre for Interdisciplinary Research in Animal Health, Faculty of Veterinary Medicine, University of Lisbon, Lisbon, Portugal; ^2^Associate Laboratory for Animal and Veterinary Sciences (AL4AnimalS), Lisbon, Portugal

**Keywords:** MDR1 mutation, herding dogs, Portugal, Portuguese breeds, pharmacotherapy

## Abstract

The impact of drug transporters in veterinary medicine has been recognized in recent years. One of the most well-characterized is the product of the *MDR1* gene, P-gp. A 4-bp deletion in the *MDR1* gene known since 2001 has been described to affect herding dog breeds. Since many used drugs in veterinary medicine are substrates for P-gp, including the macrocyclic lactones, such as avermectins, this 4-bp deletion causes a pathological condition known as “ivermectin toxicosis.” For this reason, it is important to determine the animal status concerning this mutation. In Portugal, the information of the occurrence of this mutation in our breeds is limited. The aim of the present study was to determine the occurrence of this mutation and evaluate its association with Portuguese and non-Portuguese dog breeds in Portugal. To achieve this, a total of 105 animals were studied for the presence of the *MDR1* 4-bp deletion, 23 of which were from Barbado da Terceira, 10 from Cão da Serra d'Aires, 55 belonging to breeds known to carry the mutation (Australian Shepperd, Border Collie and others) and 17 to other breeds (Labrador Retriever, Jack Russel, and others). Despite the small sample size, we observed the presence of the *MDR1* 1—delta mutation in previously described breeds and identified this mutation in Barbado da Terceira breed for the first time.

## Introduction

The impact of drug transporters on pharmacokinetics and pharmacodynamics, in veterinary medicine has been recognized over the last few years (1). One of the most well-characterized drug transporters is P-glycoprotein (P-gp,) a product of the multidrug resistance *MDR1* gene, also known as the *ABCB1* gene. In 1976, Juliano and Ling identified for the first time a deletion of 4-bp in the *MDR1* gene, the *MDR1* nt230 (del4) mutation (syn *MDR1*-1Delta, *ABCB1*-1Delta), that lead to a premature stop codon and a non-functional P-glycoprotein (2). In 2001, dogs that experienced toxic reactions following the administration of ivermectin were shown to carry the *MDR1* 1-delta mutation (3). Homozygous mutant animals (two mutant alleles [*MDR1* (–/–)]) will express a P-gp null phenotype and are 100 times more susceptible to develop neurotoxicity following ivermectin administration than dogs with the wild-type phenotype [*MDR1* (+/+)] (4, 5), and are equally sensitive to other avermectins (milbemycin, selamectin, and moxidectin) as well as to other drugs as loperamide, acepromazine, butorphanol, chemotherapy agents (vincristine, vinblastine, doxorubicin, paclitaxel), apomorphine and emodepside (4, 6–8). Heterozygous dogs [*MDR1* (+/–)], present an unpredictable phenotype concerning the reaction to these drugs implicating the need for dose adjustments (reduced doses) (5, 6).

This deletion of 4-bp in the *MDR1* gene is typically associated with shepherd breeds with studies indicating that 75% of Collies in the United States of America, France and Australia have at least one mutant allele (5). Nevertheless, the prevalence of this mutation has been reported in other breeds such as, Longhair Wippet, Australian Shepperd, Miniature Australian Shepperd, McNab Shepperd, Silken Windhound, English Shepperd, Shetland Shepperd, German Shepperd, Old English Sheepdog, and Border Collie (5, 9, 10). The animals resulting from crossbreeding of herd breeds present a frequency of ~10% and crossbred animals of 5% (6). The majority of these breeds are included in group 1 of the Federation Cynologique International (FCI) for pedigree dogs worldwide that include the sheepdogs and cattledogs breeds (11).

Since all these studies indicate that herding dog breeds have a predisposition to carry this mutation and no study has ever been conducted to evaluate the presence of the *MDR1* 1-delta mutation in Portuguese dog breeds, our main goal was to evaluate the occurrence of this mutation in dogs in Portugal, particularly in Barbado da Terceira and Cão da Serra de Aires being this last recognized as sheepdog breed in group 1 of FCI.

## Materials and methods

### Animals and sample collection

Our population sample focus on dog breeds that are known to be affected by *MDR1* 1-delta mutation and on breeds that are included in the FCI Group 1 such as two Portuguese dog breeds—Cão da Serra de Aires and Barbado da Terceira—that belong to FCI Group 1. Although Barbado da Terceira is not a recognized breed by FCI, it is classified by Clube Português de Canicultura as a Group 1 breed (12). Were also included on our population sample dog breeds that are known for not having this mutation.

For sampling purpose, clinically healthy animals were chosen. A total of 105 dogs belonging to the following breeds were sampled during the year of 2019 (from May to November): Barbado da Terceira (*N* = 23), Cão Serra de Aires (*N* = 10), Australian Shepherd (*N* = 33), Border Collie (*N* = 7), Bearded Colie (*N* = 4), Rough Collie (*N* = 4), German Shepherd (*N* = 3), Belgian Shepherd (*N* = 1), Swiss ShepherdxBorder (*N* = 8), Colie Labrador Retriever (*N* = 7), and Jack Russel (*N* = 6).

Oral swab samples were collected from Lisboa e Vale do Tejo and Sintra regions. For practical reasons samples were collected from known breeders and from dog competitions and exhibitions.

The samples were stored frozen at −80°C until analysis.

The owners provided written informed consent for the inclusion of their dogs in the study, which was approved by the Ethical Committee for Research and Education of the Faculty of Veterinary Medicine of the University of Lisbon.

### DNA extraction

The High Pure PCR Template Preparation Kit (Roche, Basel, Switzerland) was used for the extraction of the genomic DNA following the manufacturer's instructions. Briefly, the sample was mixed with binding buffer and Proteinase K and incubated at 70°C for 10 min. Then isopropanol was added, and the mixture was transferred to the column in a collection tube, centrifugated and the flow-through discarded. On the next step the inhibitor removal buffer was added, the mixture was centrifugated and the flow-through discarded. After this procedure, the wash buffer was added to the column and centrifugated and the DNA was eluted with the elution buffer.

### Polymerase chain reaction

Polymerase chain reaction (PCR) amplification followed by sanger sequencing was used for *MDR1* genotyping. Primer sequences were as follows: forward primer 5' – GGC TTG ATA GGT TGT ATA TGT TGG TG – 3'; reverse primer 5' – ATT ATA ACT GGA AAA GTT TTG TTT – 3' as previously described by Mealey and Meurs (5). For each reaction, 20 μl of the extracted genomic DNA was used in a mix containing: 0.2 pmol of Forward and Reverse primer (10 pmol), 1x of GoTaq Flexi Buffer, 2 mM of MgCl_2_ (25 mM), 0.4 mM of dNTPs, 1.5 U/μl of GoTaq Flexi DNA Polymerase (5U) (Promega, Wisconsin, USA) and MiliQ water, to a final volume of 50 μl, as a template for polymerase chain reaction (PCR) amplification under the following conditions: 94°C for 4 min; 32 cycles consisting of 94°C × 30 s, 55°C × 1 min, 72°C × 30 s; followed by 72°C for 10 min. PCR products were analyzed by 2% agarose gel electrophoresis, purified using the DNA Clean & ConcentratorTM-5 kit (Zymo Research, California, USA) following the manufacturer's instructions.

Sanger sequencing was performed by GATC using an ABI 3730XL sequencers from Applied's Biosystems. Sequence analysis was performed using the Unipro software- version 33 (Ugene).

## Results

**Figure 1:**
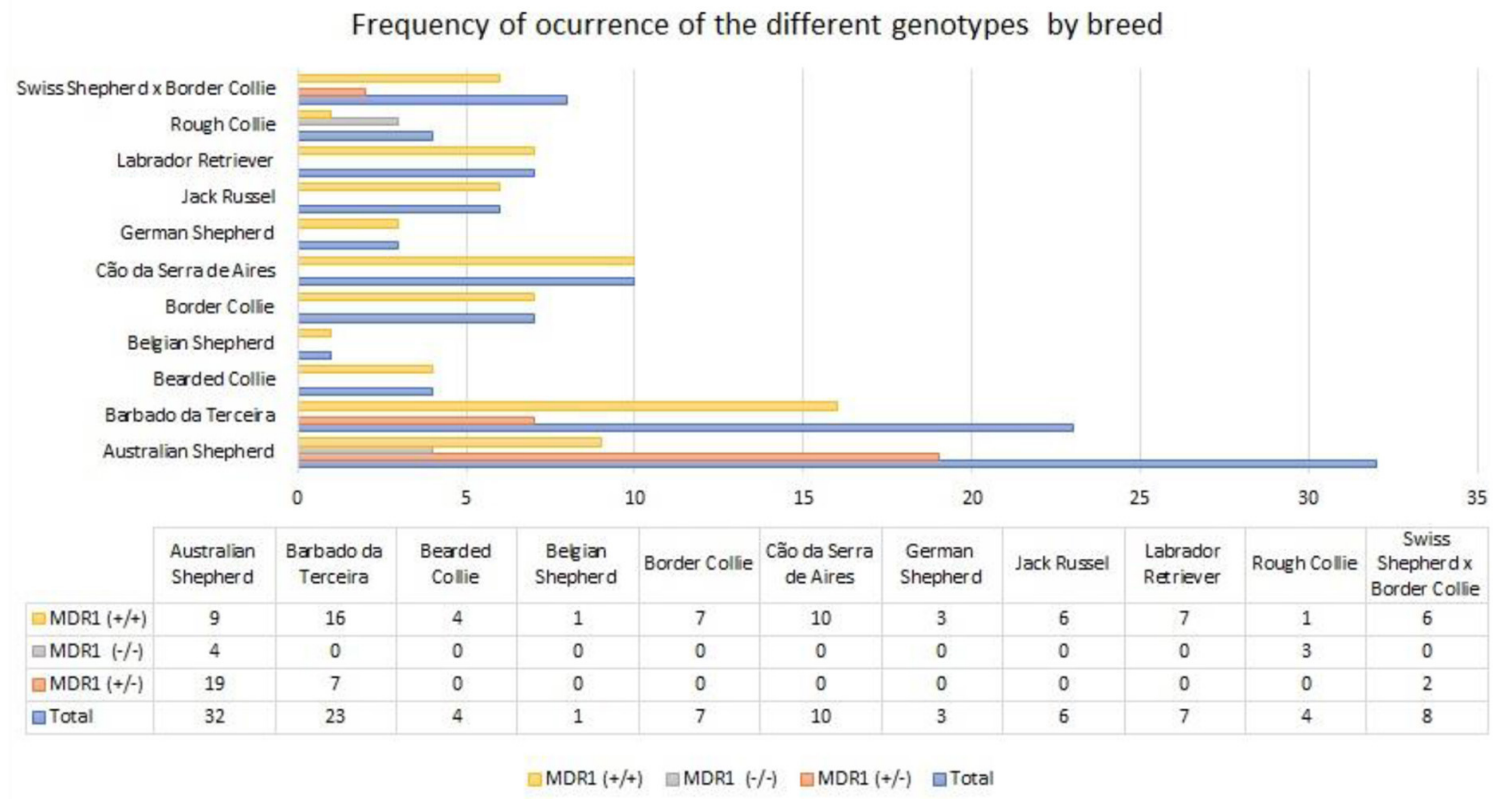
Frequency of occurrence of the different genotypes by breed. MDR1 (–/–), genotype with two mutant alleles, homozygous for the deletion, MDR1 (+/–), genotype with one wild type and one mutant allele, heterozygous for the deletion; MDR1 (+/+), genotype with two wild type alleles, homozygous.

In Figure 1, the results for the frequency of the different genotypes (homozygous mutant, heterozygous and wild type) by breed are presented. The homozygous mutant *MDR1* (–/–) genotype was only detected in Australian Shepherds (4/32) and in Rough Collies (3/4). The heterozygous *MDR1* (–/+) form was detected in Australian Shepherd (19/32), Swiss Shepherd^*^Border Collie (2/8) and Barbado da Terceira (7/23) breeds. The mutant allele was not found in the remaining genotyped breeds (Bearded Collie, Belgian Shepherd, Border Collie, Cão da Serra de Aires, German Shepherd, Jack Russel, and Labrador Retriever). In summary, 28 of 105 tested animals presented the heterozygous *MDR1* (–/+) genotype, 7/105 presented the homozygous mutant *MDR1* (–/–) genotype and all the other animals tested had the wild-type genotype.

**Figure 2:**
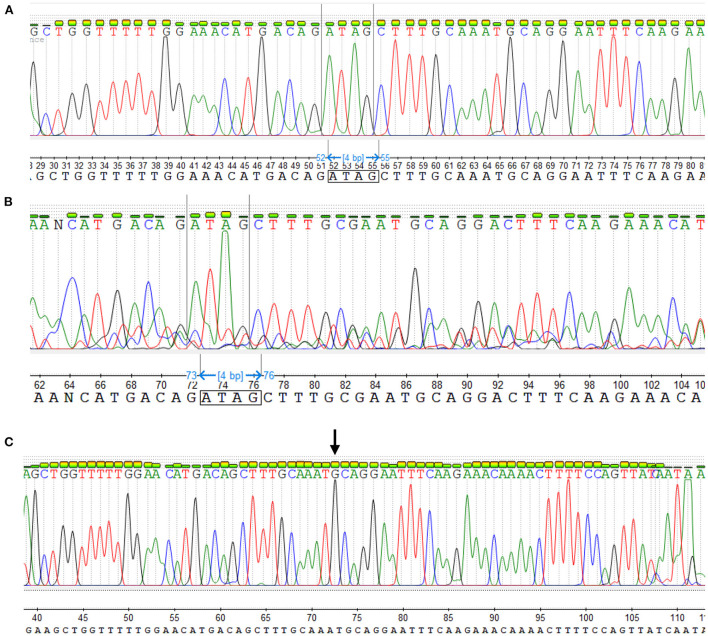
Sequence of the three genotypes. **(A)** The wild-type genotype—neither of the two alleles contains the mutation, consequently their sequence is identical. Thus, the peak of each nucleotide is tall and distinct. **(B)** Heterozygous genotype—One complete allele and one allele carrying the deletion mutation, causing distortion of the sequence output from that point (two peaks). In this way, it can be seen a C peak from de mutant sequence under the A peak from the complete allele, a bigger T peak because of the overlap between the two alleles, and a second A peak much bigger than in the wildtype sequence, also because of the overlapping. **(C)** Homozygous mutant—In this case both alleles have the mutation, thus they are identical. The arrow indicates the position where the sequence is missing.

The sequences of the three genotypes are presented on Figure 2.

## Discussion

In this study, we aimed to characterize the occurrence of the *MDR1* 1-delta mutation in herding dog breeds and Portuguese dog breeds, in Portugal. According to the CPC (Clube Português de Canicultura) official data from 2017 (13) there were a total of 36 Australian Shepherd (32/36 tested in this study), 110 Barbado da Terceira (23/110 tested in this study), 316 Belgian Shepherd (1/316 tested in this study), 61 Border Collie (7/61 tested in this study), 81 Cão da Serra de Aires (10/81 tested in this study) and 44 Rough Collie (4/44 tested in this study) registered. Thus, one of the limitations of the present study is that the sampling was not representative of the population in all breeds.

Comparing our results to *MDR1* 1-delta mutation trends from global research, the collected data agrees with previous studies, being the Australian Shepherd breed one of the most affected. Regarding this breed, an occurrence of the wild-type genotype similar to the English (14) and Australian (15) studies was obtained. The occurrence of homozygous mutant individuals was also very similar to those reported in Japanese (16) and American (5) studies. However, the occurrence of the heterozygous individuals seemed to be higher than previously reported. This discrepancy the heterozygous dog's frequency probably results from the breeding of individuals with heterozygous or an unknown genotype. Although we have a limited sample number of Border Collie, the occurrence of wildtype (100%) individuals is equal to that obtained in the United States (10) and very similar to the frequency obtained in Germany (17), Italy (18), and Thailand (19). These results probably emphasized the carefulness of the breeders, to not reproduce individuals with heterozygous and/or mutant homozygous genotypes. In relation to the breeds that do not belong to Group 1 of FCI, as expected, the mutation was not identified.

As far as we know, the assessment of the existence of this mutation in Barbado da Terceira and Cão da Serra de Aires had never been done before. Since these two breeds belong to group 1 in the FCI classification, it was possible that they would carry the *MDR1*-1delta mutation, and for drug therapeutics issues its assessment was important. In fact, we were able to detect the presence of the mutant allele, as a heterozygous genotype, in 7 of the 23 tested animals of the Barbado da Terceira breed, which, as far as we know, had not been previously reported. In contrast, the mutant allele was not detected on the Cão da Serra de Aires, but we cannot say that the mutation does not exist in this breed population as our sample does not contemplate all the existing animals of this breed in Portugal.

Barbado da Terceira is a Portuguese breed, for which origin two hypothesis have been presented (20). In the first hypothesis breed may be descendants of hunting dogs brought by colonizers from all over Europe since breeds, such as the Barbet and the Griffon, present morphological characteristics of Barbado da Terceira. A study by van Asch et al. stated that the need for sheepdogs in the Azores region led to several crosses of these breeds until they reached the Barbado da Terceira breed. This study also shows that the Azorean cattle dog has more similarities with dogs from Northern Europe than with dogs studied in Portugal mainland (21). The second hypothesis (20) states that Barbado may have descended from sheepdogs existing in European higher altitude areas, such as the Pyrenean Shepherd, the Catalan Shepherd, the Brie Shepherd, the Bergamasco, the Bearded Collie, the Bobtail, the Cattleman of the Ardennes, the Cattleman of Flanders, and the Cão da Serra de Aires. This second hypothesis is considered to be the most truthful by Cruz (22) due to the morphologic similarity and functionalities of these breeds with the Barbado da Terceira.

Due to the results in Barbado da Terceira, it is important to test the largest possible number of animals from this breed in order to obtain more accurate results. We also aim to broader the *MDR1*-1 delta mutation testing for other Portuguese breeds of sheepdogs to understand the predominance of this mutation amongst them. As stated, in the Program of Individualized Medicine (23) “The only way to know if an individual dog has the mutant *MDR1* gene is to have the dog tested” and “As more dogs are tested, more breeds will probably be added to the list of affected breeds.” As such, the information about this mutation is particularly important for breeders, not only to inform the owners about the risks and the drugs that should not be given to these animals but also to prevent the mutation from perpetuating, avoiding the matching between heterozygous animals.

Due to the small number of samples analyzed, the results must be evaluated with caution as they may not accurately represent the estimated mutation rate present in each breed. Another limitation of this study is the fact that we cannot recognize the origin of the individuals (not all of them have pedigree). Due to these limitations more studies, that include the greatest number of individuals of each breed of interest, are necessary.

## Conclusion

This study describes the occurrence of *MDR1* 1-delta mutation in herding dog breeds in Portugal (Portuguese and non-Portuguese breeds) and shows that the mutation rates in some of the breeds seems similar to those obtained in other global and local studies. This kind of information is very important and allows breeders to develop breeding strategies to reduce the incidence of this mutation in predisposed breeds and even to eliminate the mutation from genetic pools. It is important to emphasize, once more, that the clinicians should be aware of this mutation and its implications on drug therapy. Therefore, to avoid adverse reactions after administration of P-gp substrates, animals from suspected breeds should be tested as soon as possible after birth to know their genotype for *MDR1* gene.

## Data availability statement

The original contributions presented in the study are included in the article/supplementary material, further inquiries can be directed to the corresponding author/s.

## Ethics statement

The animal study was reviewed and approved by Ethical Committee for Research and Education of the Faculty of Veterinary Medicine of the University of Lisbon. Written informed consent was obtained from the owners for the participation of their animals in this study.

## Author contributions

MB, AG, SA, FA, and BS: conceptualization and writing—review and editing. MB, AG, SA, and BS: methodology. MB, AG, and SA: investigation. SA, FA, and BS: resources and funding acquisition. MB and AG: writing—original draft preparation. SA and BS: supervision. BS: project administration. All authors contributed to the article and approved the submitted version.

## Funding

This research was funded by the Fundação para a Ciência e Tecnologia (FCT), Portugal under the Project UIDB/00276/2020 for CIISA and the Project LA/P/0059/2020-AL4AnimalS.

## Conflict of interest

The authors declare that the research was conducted in the absence of any commercial or financial relationships that could be construed as a potential conflict of interest.

## Publisher's note

All claims expressed in this article are solely those of the authors and do not necessarily represent those of their affiliated organizations, or those of the publisher, the editors and the reviewers. Any product that may be evaluated in this article, or claim that may be made by its manufacturer, is not guaranteed or endorsed by the publisher.
